# Prevalence of Metabolic Syndrome and Its Components among Chinese Professional Athletes of Strength Sports with Different Body Weight Categories

**DOI:** 10.1371/journal.pone.0079758

**Published:** 2013-11-08

**Authors:** Jianjun Guo, Xi Zhang, Ling Wang, Yan Guo, Minhao Xie

**Affiliations:** 1 Centre for the Youth Sport Research and Development, China Institute of Sport Science, Beijing, China; 2 Institute of Vascular Medicine, Peking University Third Hospital, Beijing, China; 3 Department of Epidemiology & Biostatistics, School of Public Health, Peking University Health Science Center, Beijing, China; 4 Beijing Sports University, Beijing, China; University of Sao Paulo, Brazil

## Abstract

**Background:**

There is an increasing concern on cardiometabolic health in young professional athletes at heavy-weight class.

**Objective:**

Our cross-sectional survey aimed to evaluate the prevalence of metabolic syndrome and clustering of metabolic risk factors in a population of young and active professional athletes of strength sports in China.

**Methods:**

From July 2006 to December 2008, a total of 131 male and 130 female athletes of strength sports were enrolled. We used two criteria provided by the Chinese Diabetes Society (2004) and the National Cholesterol Education Program's Adult Treatment Panel III (2002) to define the metabolic syndrome and its individual components, respectively.

**Results:**

Regardless of their similar ages (mean: 21 years) and exercise levels, athletes in the heaviest-weight-class with unlimited maximum body weight (UBW) boundaries (mean weight and BMI: 130 kg and 38 kg/m^2^ for men, 110 kg and 37 kg/m^2^ for women) had significantly higher prevalence of metabolic syndrome than did those in all other body-weight-class with limited body weight (LBW) boundaries (mean weight and BMI: 105 kg and 32 kg/m^2^ for men, 70 kg and 26 kg/m^2^ for women). Prevalence of metabolic syndrome using CDS criteria (UBW vs. LBW: 89% vs. 18% for men, 47% vs. 0% for women) and its individual components, including central obesity, hypertension, hypertriglyceridemia, low high-density lipoprotein-cholesterol levels, and impaired fasting glucose, were all significantly higher in athletes at the heaviest weight group with UBW than all other weight groups with LBW.

**Conclusions:**

Our study suggests that professional athletes of strength sports at the heaviest-weight-class are at a significant increased risk of cardiometabolic disease compared with those at all other weight categories. The findings support the importance of developing and implementing the strategy of early screening, awareness, and interventions for weight-related health among young athletes.

## Introduction

In recent decades, epidemic proportions of cardiometabolic disorders, such as diabetes, hyperlipidemia, non-alcoholic fatty liver disease (NAFLD), cardiovascular and cerebrovascular diseases, become an increasing threat to public health in the general population. As role models of health and fitness, young professional athletes with regular and intensive exercise have presumably been considered as having very low risk of cardiometabolic disease. Recently, the clustering of metabolic risk factors, however, has been recognized among professional athletes [1], [2], [3]. In 2009, one research has shown that American footballers with large body sizes suffered from severe dyslipidemia and cardiometabolic disorders [4]. Furthermore, there is evidence for strength athletes with a higher risk for diabetes and metabolic syndrome after their retirement than those of other sports disciplines [5], [6], [7].

Body size has played a key role in successful performance for athletes of strength sports and American football linemen [8]. Football linemen of all ages with larger body sizes are more likely to be obese and have the metabolic syndrome and insulin resistance than their peers of other positions [9], [10]. Since there are no limits on the maximum body weight in some athletic disciplines, such as weightlifting and judo, body weights of athletes in the heaviest body weight group have become increasingly higher. At the recent 11th China national game, the average weights of weightlifting athletes at the top 3 heaviest-weight-class were 152 kg in men and 140 kg in women. Most of these athletes with large body sizes appear to be overweight or obese; however, their obesity-related health outcomes are often overlooked and under studied.

To examine cardiometabolic health of athletes at the heavy-weight-class, we conducted a cross-sectional survey among current professional athletes of strength sports in China. We aimed to 1) provide estimates of the prevalence and clustering of metabolic syndrome by sex, sport type, and different body weight groups; 2) compare the prevalence of metabolic syndrome according to CDS and NCEP ATPIII criteria; and 3) identify important demographic, lifestyle, and sports-related covariates that predict risk of metabolic syndrome among strength athletes.

## Materials and Methods

### Research Design and Methods

All active athletes at strength sports, including weightlifting, judo, wrestling, and track & field throwing (javelin, discus and shot put) athletes with extra-heavy body weight, were selected primarily from professional teams in northern provinces and the National Women's Weightlifting Team in China.

The original data collection protocols were approved by the Institutional Review Board at Sport Science, Beijing, China and written informed consents were obtained from 261 athletes.

### Participant enrollment and data collection

This cross-sectional study totally recruited 261 athletes between July 2006 and December 2008. Athletes in some of athletic disciplines, such as weightlifting and judo, are divided into several subclasses according to their body weights, of which there are no limits in the maximum body weight. An upper-limit of the heaviest-weight-class was only set for wrestling, 120 kg for men, and 72 kg for women. The participants were divided into unlimited body weight (UBW) and limited body weight (LBW) groups depending on their body weight subclasses within each of athletic disciplines. For weightlifting, judo, and track & field throwing with unlimited maximum body weight boundaries, athletes in the heaviest weight subclass were included in the UBW group and all others as the LBW group. For wrestling athletes with limited maximum body weight boundaries, since the upper weight limit of 120 kg was too heavy to be achieved, male wrestling athletes at the heaviest weight subclass were also included in the UBW group but all female athletes of wrestling were included in the LBW group.

Questionnaires of demographic, daily dietary, behavioral attitude towards obesity, routine aerobic exercises and family history of obesity for athletes were conducted. We also measured blood glucose and lipids by using overnight fasting (≥8 hours) blood samples.

Athletes were interviewed to complete the questionnaires face by face at the training bases by trained research assistants and examined by using standardized operational methods based on a standardized protocol.

### Anthropometric measurements

Physical parameters, including height, weight, waist circumference, hip circumference, sitting blood pressure, and body composition were measured. Height (in centimeter, cm), weight (in kilogram, kg), waist circumference, and hip circumference were measured without shoes, hats, coats, and sweaters. Waist circumference was measured at the midpoint between the inferior costal margin and the superior border of the iliac crest on the midaxillary line and hip circumference was measured at the maximum extension of the buttocks. Body mass index (BMI) was defined as kg/m^2^. Participants were asked to sit at ease and rest for more than 5 minutes before measurements of blood pressure. Blood pressure (BP) 30 seconds apart were measured twice from the participant's right arm, using a conventional mercury sphygmomanometer with appropriate cuff size and averaged to calculate the systolic and diastolic BP (mmHg). Hypertension was defined as BP≥140/90 mmHg or undergoing treatment for hypertension.

### Laboratory and biochemical measurements

After an overnight fast, participants sit at ease and rest for ≥ 5 minutes, and avoid smoking and drinking alcoholic beverages and coffee prior to the scheduled appointment. Venous blood samples at ≥8 hours fasting were collected into potassium oxalate/sodium fluoride anticoagulant tubes. Plasma glucose was determined by hexokinase method, with intra- and inter-assay variations less than 2.5% and 3.5%, respectively. Triglyceride (TG), high density lipoprotein cholesterol (HDL-C), low-density lipoprotein cholesterol (LDL-C) were determined by enzymatic colorimetric assay (Hitachi 7170A, Japan) with intra- and inter-assay variations less than 1.5% and 3.0%, respectively.

### Definition of metabolic syndrome

We defined the metabolic syndrome using two authoritative criteria provided by the Chinese Diabetes Society (CDS, 2004) report [11] and the Third Report of the National Cholesterol Education Program (NCEP) Expert's Panel on Detection, Evaluation, and Treatment of High Blood Cholesterol in Adults (Adult Treatment Panel III) [12]. The CDS cutoff points of metabolic syndrome components are listed as follow: waist for central obesity: men>90 cm, women>85 cm; hypertension: SBP/DBP≥135/85 mmHg; FPG≥6.1 mmol/L, and (or) 2 h PG≥7.8 mmol/L and (or) diabetes; TG≥1.7 or HDL-C<1.04 mmol/L. The NCEP ATPIII criteria includes: waist for central obesity: men>102 cm, women>88 cm; hypertension SBP/DBP≥130/85 mmHg; FPG≥6.1 mmol/L and (or) diabetes; TG≥1.7 mmol/L HDL-C men<1.04 mmol/L women<1.03 mmol/L. If the subject met 3 or more criteria, he/she was diagnosed as having metabolic syndrome.

We excluded the component of central obesity from the clusters of individual metabolic components (“≥1 MetS component” and “≥2 MetS component”). Because our strength athletes had larger body sizes than those of general population. Central obesity would be a predominant component of both “≥1 MetS component” and “≥2 MetS components”, which would raise their prevalence to reach 100% in all UBW groups. To compare the differences of other obesity-related MetS components between UBW and LBW, we therefore focused on a cluster of individual metabolic components other than central obesity.

### Statistical analysis

We first examined differences in age, anthropometric, and biochemical parameters by sex and body weight comparison groups (UBW vs. LBW). The crude means (± standard deviations) of continuous variables and count (frequency) of categorical variables were calculated. The differences between weight category groups were compared using ANOVA and logistic regression model adjusted for sex. We also compared their differences across four types of sports, including weightlifting, judo, wrestling, and track & field throwing. We then estimated the prevalence of overall and individual metabolic syndrome components according to two different criteria by sex and highest-weight class limit groups. Finally, we calculated odds ratios (ORs) and 95% confidence intervals (CIs) of the metabolic syndrome and explored potential predictors of the metabolic syndrome using logistic regression models adjusted for age and sex. We conducted a logistic regression additionally adjusted for sports type, technical class, and training time. There is a high correlation between body weight categories and NAFLD (Spearmen's rank correlation coefficient is 0.72, P<0.0001), which would provide redundant information and also lead to a collinearity problem in the logistic model. Therefore, we excluded the variable NAFLD. All P values were two-sided and statistical significance was set at 0.05 level, and all statistical analyses were conducted using SAS (version 9.2; SAS institute, Cary, NC).

## Results

Of the 261 athletes included in the study, 131 were men and 130 were women (**Table 1**). The athletes' age (mean = 21 years) and training time (mean = 7 years) between LBW group and UBW group were not significantly different. As anticipated, athletes in the UBW group were taller and heavier than those in the LBW. The means of body weight in the LBW group compared with the UBW group were 70.0 vs. 110 kg for women and 105 vs. 130 kg for men. UBW group had higher BMI (38 vs. 37 kg/m^2^ for men and 32 vs. 26 kg/m^2^ for women), waist circumference (112 vs. 105 cm and 99 vs. 69 cm), waist-hip ratio (1.04 vs. 0.92 and 0.91 vs. 0.90), and waist-height ratio (0.61 vs. 0.60 and 0.55 vs. 0.42) than the LBW group.

**Table 1:** Basic demographic, lifestyle, and biochemical characteristics by body weight categories and sex among 261 participants.

Characteristics*	LBW group (n = 160)**		UBW group (n = 101)**		P value†
	Women (n = 81)	Men (n = 79)	Women (n = 49)	Men (n = 52)	
**Age, years**	20.9±3.6	20.5±4.8	20.4±3.7	21.7±4.4	0.49
**Training time, years**	7.0 (3.0)	5.0 (4.0)	6.0 (5.0)	7.0 (3.0)	0.94
**Weight, kg**	69.9±13.0	105.4±15.8	109.9±15.2	129.6±14.2	<0.0001
**Height, cm**	164.0±8.5	181.7±8.1	173.5±4.8	184.3±6.6	<0.0001
**BMI, kg/m^2^**	25.8±3.1	31.9±3.7	36.5±4.9	38.3±4.4	<0.0001
**Waist, cm**	69.3±12.6	99.4±11.4	104.7±10.4	112.2±9.9	<0.0001
**Waist-hip ratio**	0.90±0.08	0.91±0.10	0.92±0.06	1.04±0.11	<0.0001
**Waist-height ratio**	0.42±0.06	0.55±0.06	0.60±0.06	0.61±0.06	<0.0001
**Percent body fat, %**	20.4±5.5	21.5±7.6	31.8±4.9	29.5±5.4	<0.0001
**BP, mmHg**					
SBP	110.8±10.1	121.2±9.0	124.6±15.2	137.4±13.8	<0.0001
DBP	73.6±8.2	81.5±9.0	83.8±12.0	92.2±9.6	<0.0001
**Fasting Glucose, mmol/l**	4.71 (0.55)	4.92 (0.96)	5.23 (1.20)	5.72 (1.05)	<0.0001
**Lipid, mmol/l**					
Total Cholesterol	4.6±0.8	4.6±0.8	4.7±1.2	5.5±0.9	<0.0001
Triglycerides	0.76 (0.44)	1.21 (1.30)	1.82 (1.82)	3.39 (1.78)	<0.0001
LDL-C	2.5±0.6	2.9±0.6	2.7±0.8	3.0±0.8	0.12
HDL-C	1.5±0.3	1.4±0.3	1.4±0.4	1.2±0.4	0.0002
**NAFLD, %**	2 (4.4)	17 (28.8)	43 (95.6)	42 (71.2)	<0.0001
**Technical Class, %** ††					0.001
Second class	18 (22.2)	43 (54.4)	15 (30.6)	17 (32.7)	
First class	5 (6.2)	19 (24.0)	17 (34.8)	20 (38.5)	
Master	31 (38.3)	16 (20.3)	11 (22.4)	12 (23.0)	
International Master	27 (33.3)	1 (1.3)	6 (12.2)	3 (5.8)	

* Continuous variables with normal distributions are presented as the means±standard deviations; TG, fasting glucose and training time were skewly distributed and presented as median (interquartile range); categorical variables are presented as the number (prevalence).

** Unlimited body weight (UBW): athletes in their heaviest weight class without maximum body weight limits and male athletes of wrestling with unachieved maximum weight limits; Limited body weight (LBW): all other athletes of body weight category with body weight limits.

†P value for difference between two body-weight classes were calculated by using analysis of variance or logistic regression, as appropriate, with adjustment for sex.

††P value for difference of technical classes was calculated by using chi-squares test.

NAFLD: non-alcoholic fatty liver disease was measured by ultrasonic examination.

There were significant differences in the athletes' technical classes, height, BMI, waist, waist-hip ratio, waist-height ratio, fraction of body fat, BP, fasting glucose, total cholesterol (TC) and TG by sports types. Judo athletes had the heaviest body weight. Athletes' ages or training times were not different by sports types (**Table 2**). In our study, more female track & field throwing and weightlifting athletes than male athletes were included.

**Table 2:** Anthropometric and Cardiovascular Characteristics by Heavy-Weight-Class Sports Types.

Characteristics*	Sports Types				P value†
	Weightlifting	Judo	Wrestling	Track & Field	
	n = 147	n = 46	n = 49	n = 19	
**Age, years**	20.8±3.7	21.6±4.8	20.8±5.1	19.5±3.4	0.35
**Female, n (%)**	88 (59.9)	18 (39.1)	10 (20.4)	14 (73.7)	<0.0001
**Training time, years**	7.0 (3.0)	7.0 (5.0)	4.0 (5.0)	6.0 (2.0)	0.14
**Weight, kg**	91.3±27.1	119.2±19.0	107.7±20.4	101.5±17.7	<0.0001
**Height, cm**	169.0±9.2	183.9±7.3	184.2±8.3	179.0±5.0	<0.0001
**BMI, kg/m2**	31.4±6.9	35.3±5.2	31.5±4.5	31.6±4.9	0.002
**Waist, cm**	87.6±22.8	105.3±9.3	99.7±14.6	96.5±15.0	<0.0001
**Waist-hip ratio**	0.92±0.09	0.99±0.13	0.92±0.11	0.95±0.06	0.0002
**Waist-height ratio**	0.51±0.12	0.57±0.05	0.54±0.07	0.54±0.08	0.004
**Percent body fat, %**	23.1±7.6	30.7±6.2	22.6±7.4	28.1±3.4	<0.0001
**BP, mmHg**					
SBP	118.8±15.0	130.8±14.7	120.8±9.0	126±18.6	<0.0001
DBP	79.9±11.7	87.7±10.5	81.8±9.3	79.6±13.5	0.001
**Fasting Glucose, mmol/l**	4.90 (0.87)	5.36 (1.23)	5.06 (0.99)	5.41 (1.34)	0.02
**Lipid, mmol/l**					
Total Cholesterol	4.8±1.0	5.0±1.0	4.3±0.7	5.0±1.2	0.001
Triglycerides	0.97 (2.19)	2.76 (2.74)	1.10 (0.61)	2.06 (2.16)	<0.0001
LDL-C	2.7±0.7	2.9±0.7	2.9±0.6	2.9±0.7	0.13
HDL-C	1.4±0.3	1.3±0.4	1.4±0.4	1.4±0.4	0.67
**NAFLD, %**	55 (37.4)	38 (82.6)	3 (6.1)	8 (42.1)	<0.0001
**Technical Class** **					<0.0001
Second class	39 (26.5)	18 (39.1)	32 (65.3)	4 (21.1)	
First class	28 (19.1)	19 (41.3)	7 (14.3)	7 (36.8)	
Master	45 (30.6)	9 (19.6)	9 (18.4)	7 (36.8)	
International Master	35 (23.8)	0 (0)	1 (2.0)	1 (5.3)	

* Continuous variables with normal distributions are presented as the means±standard deviations; TG, fasting glucose and training time were skewly distributed and presented as median (interquartile range); categorical variables are presented as the number (prevalence).

†P value for difference across four types of sports were calculated by using analysis of variance or Chi-square test, as appropriate.

** P value for difference of technical classes was calculated by using Fisher's Exact Test.

NAFLD: non-alcoholic fatty liver disease which was measured by ultrasonic of liver.

**Figure 1:**
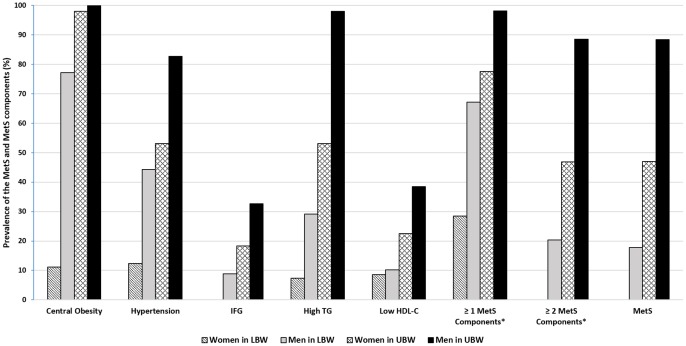
The prevalence of metabolic syndrome and its components were stratified by sex and two body weight comparison groups. Metabolic syndrome and its individual factors were defined by CDS 2004. The cutoff points are as follow: waist for central obesity: men >90 cm, women >85 cm; hypertension: SBP/DBP≥135/85 mmHg; FPG≥6.1 mmol/L, and (or) 2 h PG≥7.8 mmol/L and (or) diabetes; TG≥1.7 or HDL-C<1.04 mmol/L. *Central obesity was excluded from the “≥1 MetS Components” and “≥2 MetS Components” in order to compare the differences of other obesity-related MetS components given very high prevalence of central obesity. MetS: Metabolic syndrome; UBW (Unlimited body weight): athletes in the heaviest weight category without maximum body weight limits and male athletes of wrestling with unachieved maximum weight limits; LBW (Limited body weight): all other athletes of body weight category with body weight limits.

As compared with women in the LBW, active professional athletes in other groups (women in the UBW group and men in both LBW and UBW groups) had significantly higher proportions of central obesity, hypertension, hypertriglyceridemia, dyslipidemia (high TG and low HDL-C), and the metabolic syndrome (**Figure 1**). Prevalence of metabolic syndrome using CDS criteria and its individual components, including central obesity, hypertension, hypertriglyceridemia, low high-density lipoprotein-cholesterol levels, and impaired fasting glucose were predominantly higher in athletes at the heaviest weight group with UBW than all other weight groups with LBW.

Prevalence of central obesity component of metabolic syndrome was very high in our athletes' population. The prevalence of central obesity (CDS criteria) defined by waist circumference was 100% for male and 98% for female in the UBW group and 77% and 11% for male and female in the LBW group, respectively. According to the BMI criterion for defining obesity and overweight in Chinese adults, a BMI of 24.0–27.9 kg/m^2^ is the criterion for overweight and ≥28 kg/m^2^ is for obesity. Thus, 98% athletes (100% in men, 95.9% in women) in the UBW group were considered overall obesity as opposed to 48.8% (82.3% in men, 16.0% in women) in the LBW group.

Similarly, prevalence of other individual metabolic syndrome components using CDS criteria, including hypertension (83% vs. 44% for men and 53% vs. 12% for women), hypertriglyceridemia (98% vs. 29% and 53% vs. 7%), and low HDL-C levels (38% vs. 10% and 22% vs. 9%) were much higher in the athletes of UBW group than in those of LBW group. Their prevalence were similarly high if ATPIII criteria was used (data not shown). By contrast, they, with the exception of women in the LBW group (0%), had high fasting glucose levels with relatively small proportions of 8–33%.

Collectively, athletes in the UBW group had significantly higher prevalence of the metabolic syndrome components than those in the LBW group for women and men. Overall, more than 72% of all participants had at least one of metabolic syndrome components and 63% of all participants had at least one of metabolic syndrome components except the central obesity. According to the diagnostic standards of metabolic syndrome defined by the CDS, the prevalence of metabolic syndrome of all athletes was 31.8%, of which the prevalence in the UBW group was significantly higher than that in the LBW group (49% vs. 0% for women and 88% vs. 18% for men).

In the univariate logistic regression model, sex, sports type, body weight group, and fatty liver disease were significantly associated with the prevalence of the metabolic syndrome, regardless of definition criteria. After adjusted for sex and age, the ORs of metabolic syndrome diagnosed by CDS criteria (95% CI) were 0.02 (0.007, 0.05) for the upper weight limit group (LBW vs. UBW), 0.17 (0.04, 0.65) for sports type (wrestling vs. track & field), 3.44 (1.60, 7.37) for technical class (first class vs. second class), and 17.7 (8.1, 38.6) for nonalcoholic fatty liver disease (moderate and severe vs. none). If ATPIII criteria was used, the only change was sports type; the OR was also significant for judo as compared with track & field (**Table 3**).

**Table 3:** Odds ratios (ORs) and 95% confidence intervals (CIs) of metabolic syndrome by demographical and lifestyle risk factors among 261 participants.

Factors	CDS	ATPIII	CDS	ATPIII
	No. of cases/controls	No. of cases/controls	OR (adjusted 95% CI)†	OR (adjusted 95% CI)†
**Age**, years	83/178	79/182	1.08 (1.01, 1.15)	1.08 (1.01, 1.15)
**Sex**				
female	23/107	23/107	0.26 (0.14, 0.45)	0.29 (0.16, 0.51)
male	60/71	56/75	1	1
**Body weight group** *				
LBW group*	14/146	9/151	0.02 (0.007, 0.05)	0.01 (0.003, 0.030)
UBW group*	69/32	70/31	1	1
**Sports type**				
Weightlifting	39/108	35/112	0.53 (0.17, 1.65)	0.63 (0.20, 2.02)
Judo	29/17	31/15	2.16 (0.62, 7.51)	3.74 (1.04, 13.42)
Wrestling	9/40	8/41	0.17 (0.04, 0.65)	0.21 (0.05, 0.86)
Track & Field	6/13	5/14	1	1
**Technical Class**				
Second class	22/71	24/69	1	1
First class	33/28	31/30	3.44 (1.60, 7.37)	2.28 (1.09, 4.80)
Master	24/46	20/50	1.42 (0.55, 3.72)	0.63 (0.24, 1.70)
International Master	4/33	4/33	0.46 (0.11, 1.85)	0.25 (0.06, 1.03)
**Training time**				
5 years or less	29/68	29/68	1	1
More than 5 years	54/110	50/114	1.15 (0.59, 2.25)	0.93 (0.48, 1.83)
**NAFLD**				
None	20/137	17/140	1	1
Mild	19/20	18/21	6.47 (2.79, 14.96)	6.90 (2.95, 16.16)
Moderate & Severe	44/21	44/21	17.65 (8.07, 38.60)	20.52 (9.27 45.38)

* Unlimited body weight (UBW): athletes in their heaviest weight class without maximum body weight limits and male athletes of wrestling with unachieved maximum weight limits; Limited body weight (LBW): all other athletes of body weight category with body weight limits.

†Multiple logistic regression model adjusted for age, sex; model for age and sex was adjusted for each other.

NAFLD: non-alcoholic fatty liver disease was measured by ultrasonic examination.

The results of the multi-variable model adjusted for sports type, technical class and training time were slightly different from the results of age- and sex-adjusted model. The body weight category was still a statistically significant risk factor for metabolic syndrome defined by CDS criteria (P<0.0001, RR = 0.007 [95%CI: 0.007, 0.029]) or ATPIII criteria (P<0.0001, RR = 0.002 [95%CI: 0.0002, 0.015]). We also found that the age was not a significant risk factor of metabolic syndrome (P = 0.997 for CDS criteria, P = 0.519 for ATPIII criteria). Sex and sports type were still significant for metabolic syndrome defined by ATPIII criteria (P<0.0001 for sex, P = 0.016 for sports type), but sports type was marginal significant when CDS criteria (P = 0.06) was used.

## Discussion

Our cross-sectional study showed that cardiometabolic risk factors were predominantly prevalent among Chinese professional athletes at the heaviest-weight classes, despite their young ages and similar levels of training time. Consistent with previous findings in professional athletes engaged in strength-related sports, our study showed that these young active athletes with large body sizes were not as healthy as we expected and indeed at high risk for cardiometabolic disease. Our athletes, either women or men, had large body sizes, with an average body weight of 115.0 kg in men and 85.0 kg in women and BMI of 34.4 kg/m^2^ in men and 29.8 kg/m^2^ in women. Our study showed that larger body sizes were related to higher prevalence of metabolic syndrome. Compared with American football players, our study population had relatively lower body weights but a high prevalence of the metabolic syndrome (45.8% in men and 17.7% in women). A survey of 70 American football players in 2008 revealed that 34 (48.6%) athletes suffered from metabolic syndrome [5]. The average height of athletes' was 187.5 cm, the mean body weight was over 120 kg. And the waist of the athletes with metabolic syndrome were all larger than 102 cm [5]. As shown in another study of American football players, the average age of these players were only 19.9 years old but their risk of getting diabetes and cardiovascular diseases was much higher than that of the general population [9].

BMI is a common method used worldwide as a standard measurement of overall adiposity in general adults. The BMI standard is less affected by the height; however, it does not distinguish the muscle-related body weight to fat-related weight, which overestimates the degree of obesity in muscular athletes. The BMI cutoff of 24 kg/m^2^ was proposed to define overweight in Chinese adults [13].

In the 2006 version of “Guidance for the Prevention and Control of Overweight and Obesity in China” [14], it is advised that using both BMI and body fat percentage provided more accurate measurements of the degree of obesity, especially for muscular athletes. In 2008, a study on American football players [9] showed that 35 kg/m^2^ would be an effective threshold for American football players based on the presence of left ventricular hypertrophy. However, it remains to be determined whether this cutoff levels is applicable for obesity definition among Chinese athletes.

Abdominal fat is mainly the abdominal subcutaneous fat and visceral fat, which is also called central obesity. In fact, there are no standards existing for the diagnosis of central obesity for athletes. According to the NCEP-ATPIII criteria, male waist circumference over 102 cm or female waist circumference over 88 cm is classified as central obesity. Based on this standard, 68 of 80 UBW male athletes (86.5%) and 47 of 49 female athletes (95.9%) had central obesity in the current analysis. According to the “Guide on Prevention and Treatment of Overweight and Obesity of Chinese Adults” [14], the cutoff point of central obesity for male is ≥ 85 cm and ≥ 80 cm for female, the prevalence of central obesity would be even higher. Since long term training has significantly impact on fat distribution, the cutoff levels of central obesity may be different between athletes with high levels of exercise and large body size and general adults.

High TG and low HDL-C have been reported as the main phenotypic feature of dyslipidemia, although most people with dyslipidemia in China suffer from hypertriglyceridemia [15]. The prevalence of hypertriglyceridemia in people over 18 years old is 11.9%, 14.5% for male and 9.9% for female. The second common syndrome is low blood HDL cholesterol with the prevalence of 7.4%, followed by hypercholesterolemia of 2.9% [16]. In this study, dyslipidemia was common among athletes at the heaviest-weight class. The prevalence of hypertriglyceridemia in the UBW athletes is higher than that of ordinary people. The prevalence of low HDL-C level was 17.2%, which was again much higher than that in the general population (7.4%). Among the 81 female LBW athletes, 46.9% had low HDL-C levels but only 9.9% had high TG levels. As the hypertension definition defined by 2011 revision of China's prevention and cure guide of hypertension, mild hypertension was defined as SBP: 140∼159 mmHg and/or DBP: 90∼99 mmHg, moderate hypertension was SBP: 160∼179 mmHg and/or DBP: 100∼109 mmHg, sever hypertension was SBP≥180 mmHg and/or DBP≥110 mmHg. The overall prevalence of hypertension within the UBW athletes was 55.4%, 49.5% of the UBW athletes had mild and moderate hypertension, and 5.9% of them developed severe hypertension. Insulin resistance as reflected by high fasting glucose was less common in the LBW group than in the UBW group.

As a constellation of metabolic abnormalities, metabolic syndrome is a major determinant or precursor of cardiometabolic disease, although there is as yet no consensus regarding the definitive criteria for different ethnic populations [17]. This study shows that the prevalence of metabolic syndrome in the studied athletes was 31.8%. Of the UBW men and women, 47%, 88% and 47%, 90% had the metabolic syndrome under the CDS and the NCEP ATPIII criteria, respectively. Of the LBW men and women, they were 18%, 0% and 11%, 0%. The prevalence of metabolic syndrome in our active athletes were slightly higher than 12% observed by previously study of Chinese adults in 2004–2005 using CDS criteria [18] and 18% reported using the NCEP ATPIII criteria in Shanghai men's Health Study [19]. However, the different cutoffs used in CDS or NCEP ATPIII criteria may not appreciate to young and active athletes.

Several limitations of the current study merit consideration. First, our study was cross-sectional by design and cannot address a causal relation of adiposity and metabolic abnormalities. We cannot further assess the natural history of adiposity and its metabolic consequences. We also cannot determine the cutoff levels for adiposity or central obesity for active athletes with large body size without longitudinal data. We cannot exclude the potential confounding from dietary factor due to limited detailed information using food frequency questionnaire. However, the possibility should be low because the accommodations and foods were uniformly and freely provided by governmental funding and diets outside of the facilities are strictly forbidden for these active professional athletes. In addition, the findings in this population with large body sizes may not be generalizable to other athlete populations.

In conclusion, this study suggests that young professional athletes at the heaviest-weight class had significantly increased prevalence of metabolic risk factors as comparing with those in all other weight categories. Consistent with previous findings among professional athletes engage in other strength-related sports, our results support the importance of developing effective strategies of early screening, awareness, and intervention of obesity-associated health outcomes among young athletes, especially those at the heavy-weight class.
